# Chloroplast genome features of *Moricandia arvensis* (Brassicaceae), a C3-C4 intermediate photosynthetic species

**DOI:** 10.1371/journal.pone.0254109

**Published:** 2021-07-08

**Authors:** Bin Zhu, Lijuan Hu, Fang Qian, Zuomin Gao, Chenchen Gan, Zhaochao Liu, Xuye Du, Hongcheng Wang

**Affiliations:** School of Life Sciences, Guizhou Normal University, Guiyang, People’s Republic of China; Chinese Academy of Medical Sciences and Peking Union Medical College, CHINA

## Abstract

*Moricandia arvensis*, a plant species originating from the Mediterranean, has been classified as a rare C3-C4 intermediate species, and it is a possible bridge during the evolutionary process from C3 to C4 plant photosynthesis in the family Brassicaceae. Understanding the genomic structure, gene order, and gene content of chloroplasts (cp) of such species can provide a glimpse into the evolution of photosynthesis. In the present study, we obtained a well-annotated cp genome of *M*. *arvensis* using long PacBio and short Illumina reads with a *de novo* assembly strategy. The *M*. *arvensis* cp genome was a quadripartite circular molecule with the length of 153,312 bp, including two inverted repeats (IR) regions of 26,196 bp, divided by a small single copy (SSC) region of 17,786 bp and a large single copy (LSC) region of 83,134 bp. We detected 112 unigenes in this genome, comprising 79 protein-coding genes, 29 tRNAs, and four rRNAs. Forty-nine long repeat sequences and 51 simple sequence repeat (SSR) loci of 15 repeat types were identified. The analysis of Ks (synonymous) and Ka (non-synonymous) substitution rates indicated that the genes associated with “subunits of ATP synthase” (*atpB*), “subunits of NADH-dehydrogenase” (*ndhG* and *ndhE*), and “self-replication” (*rps12* and *rpl16*) showed relatively higher Ka/Ks values than those of the other genes. The gene content, gene order, and LSC/IR/SSC boundaries and adjacent genes of the *M*. *arvensis* cp genome were highly conserved compared to those in related C3 species. Our phylogenetic analysis demonstrated that *M*. *arvensis* was clustered into a subclade with cultivated *Brassica* species and *Raphanus sativus*, indicating that *M*. *arvensis* was not involved in an independent evolutionary origin event. These results will open the way for further studies on the evolutionary process from C3 to C4 photosynthesis and hopefully provide guidance for utilizing *M*. *arvensis* as a resource for improvinng photosynthesis efficiency in cultivated *Brassica* species.

## Introduction

*Moricandia arvensis* (Brassicaceae) originates from the Mediterranean and is mainly distributed in the Mediterranean Basin, North Africa, and west and southeast Asia [1]. *M*. *arvensis* is commonly used as an ornamental garden flower because of its vivid violet purple petals. Additionally, *M*. *arvensis* leaf extracts have strong antioxidant and antigenotoxic effects [2], making this species a suitable nutraceutical research model. Moreover, interspecific hybridization between *M*. *arvensis* and cultivated *Brassica juncea* demonstrated that *M*. *arvensis* can be used as an important cytoplasmic male sterility resource for *B*. *juncea* [3]. Furthermore, somatic hybridization between *M*. *arvensis* and *B*. *juncea* has been achieved to develop a novel cytoplasmic male sterility (CMS) system and restore *B*. *juncea* lines [4, 5].

Intriguingly, despite the fact that the family Brassicaceae does not contain any C4 species, *M*. *arvensis* has been identified as a C3-C4 intermediate species based on gas exchange parameters, leaf anatomy, metabolome and transcriptome [6–8], making it a desirable resource for introducing an intermediate C3-C4 photosynthetic phenotype into cultivated *Brassica* species. Interspecific hybridization between *M*. *arvensis* and cultivated *Brassica* crops employing ovary and ovule rescue methods has been extensively conducted for decades to transfer the C3-C4 photosynthetic character and drought tolerance traits [9–11]. The C3-C4 intermediate stage is believed to be a transition state between the evolutionary history of C3 and C4 plants [8, 12]. Therefore, it is of great interest to use *M*. *arvensis* to study photosynthetic evolution [8, 13]. A recent study employing C3-C4 intermediate, C4, C4-like, and C3 photosynthetic species within the genus *Flaveria* demonstrated that the RLSB (the nuclear-encoded *rbcL* RNA S1 binding domain protein), the only mRNA-binding protein associated with cp *rbcL* gene regulation, is likely involved in the evolution of C3-C4 photosynthesis [14]. Moreover, the cp *rbcL* gene encodes eight large subunits of Rubisco [15], which is a key enzyme involved in the first major step of carbon fixation. Given the indispensable role of chloroplasts in plant photosynthesis, deciphering the cp genome features of a C3-C4 intermediate plant could offer a glimpse into the evolution of photosynthesis.

The cp genome generally shows a typical quadripartite cycle (120–160 kb in size), harboring 110–130 genes [16, 17]. In most angiosperms, this quadripartite circular structure comprises of a large single copy (LSC) region and a small single copy (SSC) region, which are separated by a pair of inverted repeats (IR) regions [18]. The cpDNA evolution rate is much slower than that of nuclear DNA [19, 20] because of fewer recombination events, lower nucleotide replacement rates, and predominantly maternal inheritance of the cp genome. Therefore, cpDNA has been widely employed to decipher the genealogical relationships among plant species [21–23]. Previous, phylogenetic studies that used several nuclear/cp genes (sequences) have demonstrated that C3-C4 intermediate species generally have closer relationships with C4 relatives than with C3 relatives [24]. Studies based on cytological analysis [25, 26] and restriction site analysis [27] have revealed that *M*. *arvensis* is closely related to cultivated *Brassica* species. However, one study that carried out a genetic analysis of the *S*-locus reported a contrary result; it indicates that *Moricandia* species had a distant relationship with *Brassica* species [28].

In the present study, we obtained and fully described the complete cp genome of *M*. *arvensis* through *de novo* assembly based on long PacBio reads and short Illumina reads. To determine whether the C3-C4 intermediate *M*. *arvensis* evolved in an independent path among Brassicaceae species, we used the cp genomes of 59 other Brassicaeae species downloaded from GenBank to determine the genealogical relationship between *M*. *arvensis* and these species. Our results will open the way for further studies on the evolutionary path from C3 to C4 photosynthesis and hopefully provide guidance for utilizing *M*. *arvensis* as a resource to improve photosynthesis in cultivated *Brassica* species.

## Materials and methods

### Ethical statement

This study did not involve in any human or animal research participant data. The plant sample tested in this study is not endangered species, and the collection of sample didn’t cause any environmental problem.

### Plant materials and DNA library preparation

Seeds from a pure *M*. *arvensis* line were cultivated in a glasshouse at Guizhou Normal University (Guiyang, China). When the plants had seven true leaves, 5 g of fresh leaves were collected for total DNA isolation using a commercial DNA extraction kit (TIANGEN, KG203, Beijing) according to the manufacturer’s instructions. After checking the integrity of the DNA with the Agilent Technologies 2100 Bioanalyzer (Agilent Technologies, USA), ~1 μg DNA was fragmented to ~450 bp to construct a short-insert library for Illumina sequencing (HiSeq X Ten). Approximately 5 μg of DNA were used to construct the library with insert sizes of 20 kb for PacBio sequencing (PacBio, Menlo Park, USA), according to the manufacturer’s instructions. The raw sequence data reported in this paper were deposited in the Genome Sequence Archive of the National Genomics Data Center under the accession number CRA003542 (https://bigd.big.ac.cn/search/?dbId=gsa&q=CRA003542).

### Cp genome assembly and genome feature analysis

The Illumina platform produced 150 bp of paired-end reads. Then, the Trimmomatic software (version-0.39) with default settings was used to remove low-quality reads and trim out the adapters to obtain clean reads [29]. To identify the cp-related reads, the clean reads were mapped to the published *Arabidopsis thaliana* cp genome (NC_000932) using the BLASR software under basic local alignment with default settings [30]. The cp-related reads were combined into contigs using the SOAPdenovo software (version 2.04) with default parameters [31]. After removing the debased PacBio reads with read quality < 0.80 or read length < 500 bp, long PacBio subreads were used to repair the gaps between contigs using PBjelly [32]. Then, the BWA software (version 0.5.9) was employed to correct possible misassembly and errors throughout these cp-related Illumina reads [33]. Finally, the frameshift errors were manually corrected during gene prediction.

GeSeq (https://chlorobox.mpimp-golm.mpg.de/geseq.html/) was used to annotate *M*. *arvensis* cp genome using default settings. Basic Local Alignment Search Tool (BLAST) was used to define the start and stop codons of each gene through homology searches [34]. The complete gene map of the *M*. *arvensis* cp genome was plotted using the OGDraw software (version 1.2) [35]. Finally, the well-annotated *M*. *arvensis* cp genome was submitted to public GenBank under the accession number MW279233.

### Long repeat and simple sequence repeat (SSR) analysis

REPuter (https://bibiserv.cebitec.uni-bielefeld) [36] was used to detect the long repeat sequences in the *M*. *arvensis* cp genome with the default settings: minimal repeat size, 30; sequence consistency, > 90%; maximum computed repeats, 50. The MISA (https://webblast.ipk-gatersleben.de/misa/) [37] software was used to detect the SSR loci with the following settings: 10 repeats for mono- unit, five repeats for di- unit, four repeats for tri-units, and three repeats for tetra-, penta-, and hexa- units.

### Codon usage bias of the coding sequences

Codon usage bias is believed to affect translational dynamics, including protein folding, translation accuracy, and efficiency [38]. The CodonW1.4.2 program (http://downloads.fyxm.net/CodonW-76666.html/) with default settings was used to study the translational dynamics of the *M*. *arvensis* cp genome [39].

### Comparison of the *M*. *arvensis* cp genome with C3 cp genomes of other Brassicaceae species

To detect sequence divergence between the *M*. *arvensis* cp genome and other related C3 species, the mVISTA web service was used to visualize genome divergence, using the *M*. *arvensis* cp genome as a reference. The related cp genome sequences included *B*. *rapa* (NC_040849), *B*. *oleracea* (NC_041167), *B*. *juncea* (NC_0282720), *Raphanus sativus* (NC_024469), and *Orychophragmus diffuses* (NC_033498), and they were downloaded from the NCBI. Additionally, IRscope was used to compare the LSC/IRB/SSC/IRA junction regions among the selected cp genomes.

### Ks/Ka substitution rate calculation

Synonymous (Ks) and non-synonymous (Ka) nucleotide substitution rates are valuable markers for evaluating genomic evolution [40, 41]. To calculate the Ks and Ka ratios, total pairwise comparisons of 77 common shared coding genes (S1 Table) among the selected Brassicaceae cp genomes (*B*. *rapa*, *B*. *oleracea*, *B*. *juncea*, *O*. *diffuses*, *R*. *sativus*, and *M*. *arvensis*), were determined using the KaKs calculator (version 2.0) with default parameters [42]. Pairwise alignments of these genes were carried out using MAFFT [43] with default settings.

### Phylogenetic analysis

The maximum likelihood (ML) method with the Tamura-Nei model which was automatically recommended by the MEGA7 was used to determine the genealogical relationship between *M*. *arvensis* and related Brassicaceae species [44]. The initial tree for the heuristic search was obtained automatically by applying the Neighbor-Join and BioNJ algorithms to a matrix of pairwise distances estimated by the Maximum Composite Likelihood (MCL) approach. A total of 60 cp Brassicaceae species genomes (S2 Table) were downloaded from GenBank to construct the phylogenetic trees. To increase the efficiency of the phylogenetic analysis, 66 homologous coding sequences (S3 Table) shared by the studied cp genomes were used. A total of 1,000 bootstrap replications were used to increase confidence.

## Result

### The features of *M*. *arvensis* cp genome

A total of 6,834 Mb of raw data (45,560,522 raw reads) were obtained from the Illumina sequencing platform. After filtering, 6,543.7 Mb of clean data (43,846,822 clean reads) were obtained with an average Q20 value of 98.46%. A total of 3,530 PacBio subreads with a mean length of 12,382 bp were obtained (S4 Table). After being mapped to the *Arabidopsis thaliana* cp genome, 482.3 Mb (7.37% of clean reads) cp-related reads was obtained, representing an coverage of 3,306× over the cp genome. And 187 PacBio subreads (5.3% of total PacBio subreads) were demonstrated to cp-related subreads. Because of the relatively low coverage of the cp genome, these cp-related PacBio subreads were only used to fill the gaps between the contigs constructed by the Illumina reads.

**Fig 1:**
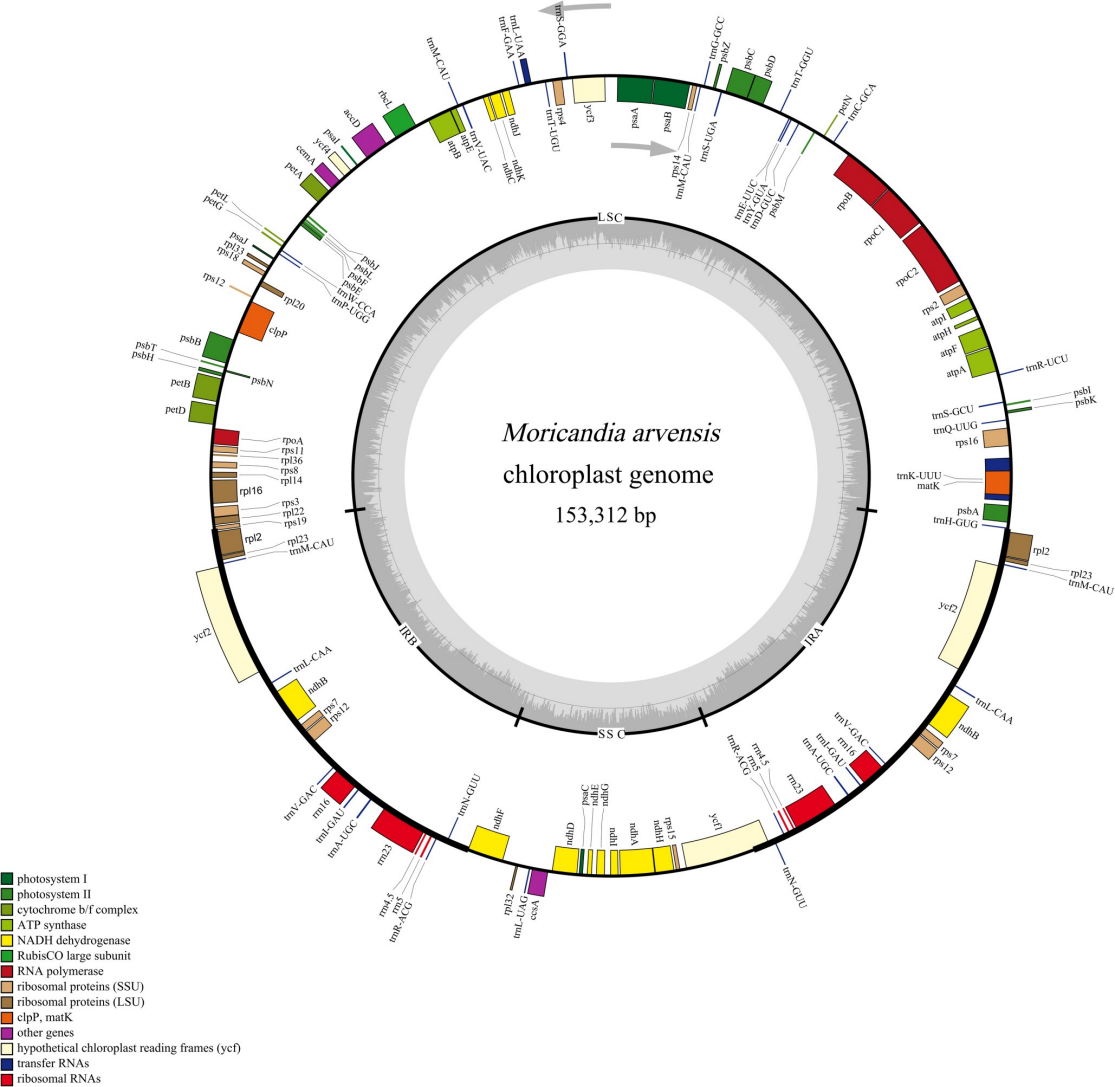
Gene map of the complete *M*. *arvensis* cp genome. Genes on the outside and inside of the circle are transcribed in clockwise and counterclockwise directions, respectively. Genes belonging to different functional groups are color coded. Color intensity refering to the inner circle corresponds to GC content. The SSC, LSC, and inverted repeat regions (IRA and IRB) are indicated.

The cp genome of *M*. *arvensis* had a quadripartite structure of 153,312 bp in size, including an SSC region of 17,786 bp and an LSC region of 83,134 bp, which were separated by two IR regions of 26,196 bp (Table 1 and Fig 1). Its average GC content was 36.37%, and the IR region had the highest GC content (42.34%), followed by LSC (34.15%), and SSC (29.17%). The *M*. *arvensis* cp genome encoded 112 genes, including four rRNAs, 29 tRNAs, and 79 protein-coding genes. Among these genes, 20 genes (four rRNAs, eight tRNAs, and eight protein-coding genes) were duplicated. The coding sequence region was 78,396 bp in size, accounting for 51.13% of the whole genome. Among these genes, 82 genes, including 59 coding genes and 23 tRNAs, were identifed in the LSC region. Twelve genes were observed in the SSC region, whereas 18 genes, including seven tRNAs, four rRNAs, and seven protein coding genes, were duplicated in the IR regions. In the whole genome, 12 genes (four tRNAs and eight coding genes) had one intron, whereas two genes (*ycf3* and *clpP*) had two introns (Table 2). The *rps12* was identified to be a trans-spliced gene harboring one intron.

**Table 1:** The detail characteristics of the complete cp genome of *Moricandia arvensis*.

Category	Items	Descriptions
Construction of cp genome	LSC region (bp)	83134
	IRA region (bp)	26196
	SSC region (bp)	17786
	IRB region (bp)	26196
	Genome Size (bp)	153,312
Gene content	Total genes	132
	Protein-coding genes	87
	tRNAs	37
	rRNAs	4
	Two copy genes	20
	Genes on LSC region	84
	Genes on IRA region	18
	Genes on SSC region	12
	Genes on IRB region	18
	Gene total length (bp)	78,396
	Average of genes length (bp)	933
	Gene length / Genome (%)	51.13
GC content	GC content of LSC region (%)	34.15
	GC content of IRA region (%)	42.34
	GC content of SSC region (%)	29.17
	GC content of IRB region (%)	42.34
	Overall GC content (%)	36.37

**Table 2:** Summary of assembled gene functions of *Moricandia arvensis* cp genome.

Category for genes	Groups of genes	Name of genes
Genes involvingin photosynthesis	Subunits of photosystem	*psaA*, *psaB*, *psaC*, *psaI*, *psaJ*, *psbA*, *psbB*, *psbC*, *psbD*, *psbE*, *psbF*, *psbH*, *psbI*, *psbJ*, *psbK*, *psbL*, *psbM*, *psbN*, *psbT*,*psbZ*
	Subunits of cytochrome b/f complex	*petA*, *petB*, *petD*, *petG*, *petL*, *petN*
	Large subunit of Rubisco	*rbcL*
	Subunits of ATP synthase	*atpA*, *atpB*, *atpE*, *atpF*^*b*^,*atpH*, *atpI*
	Subunits of NADH-dehydrogenase	*ndhA*^*b*^, *ndhB*^*a*,*b*^, *ndhC*, *ndhD*, *ndhE*, *ndhF*, *ndhG*, *ndhH*, *ndhI*, *ndhJ*, *ndhK*
Self-replication	Ribosomal RNA genes	*rrn16*^*a*^, *rrn23*^*a*^, *rrn4*.*5*^*a*^, *rrn5*^*a*^
	Transfer RNA genes	*trnA-UGC*^*a*,*b*^, *trnC-GCA*, *trnD-GUC*, *trnE-UUC*, *trnF-GAA*, *trnG-GCC*, *trnH-GUG*, *trnI-GAU*^*a*,*b*^, *trnK-UUU*^*b*^, *trnL-CAA*^*a*^, *trnL-UAA*, *trnL-UAG*, *trnM-CAU*^*a*^, *trnN-GUU*^*a*^, *trnP-UGG*,*trnQ-UUG*, *trnR-ACG*^*a*^, *trnR-UCU*, *trnS-GCU*, *trnS-GGA*, *trnS-UGA*, *trnT-GGU*, *trnT-UGU*, *trnV-GAC*^*a*^, *trnV-UAC*^*b*^, *trnW-CCA*, *trnY-GUA*, *trnG-UCC*, *trnI-CAU*^*a*^
	Small subunit of ribosome	*rps11*, *rps12*^*a*,*b*^, *rps14*, *rps15*, *rps16*^*b*^, *rps18*, *rps19*, *rps2*, *rps3*, *rps4*, *rps7*^*a*^, *rps8*,
	Large subunit of ribosome	*rpl14*, *rpl16*^*b*^, *rpl2*^*a*,*b*,^ *rpl20*, *rpl22*, *rpl23*^*a*^, *rpl32*, *rpl33*, *rpl36*,
	DNA-dependent RNA polymerase	*rpoA*, *rpoB*, *rpoC1*^*b*^, *rpoC2*
Other genes	Maturase	*matK*
	Envelope membrane protein	*cemA*,
	Subunit of acetyl-CoA	*accD*
	C-type cytochrome synthesis gene	*ccsA*
	Protease	*clpP*^*c*^
Functionally unknown genes	Conserved Open reading frames	*ycf1*^*a*^, *ycf2*^*a*^, *ycf3*^*c*^, *ycf4*, *ycf15*^*a*^

^a, b, c^ The letters indicate the gene with two copes, harboring one intron and two introns, respectively.

### Comparisons of whole cp genomes

**Fig 2:**
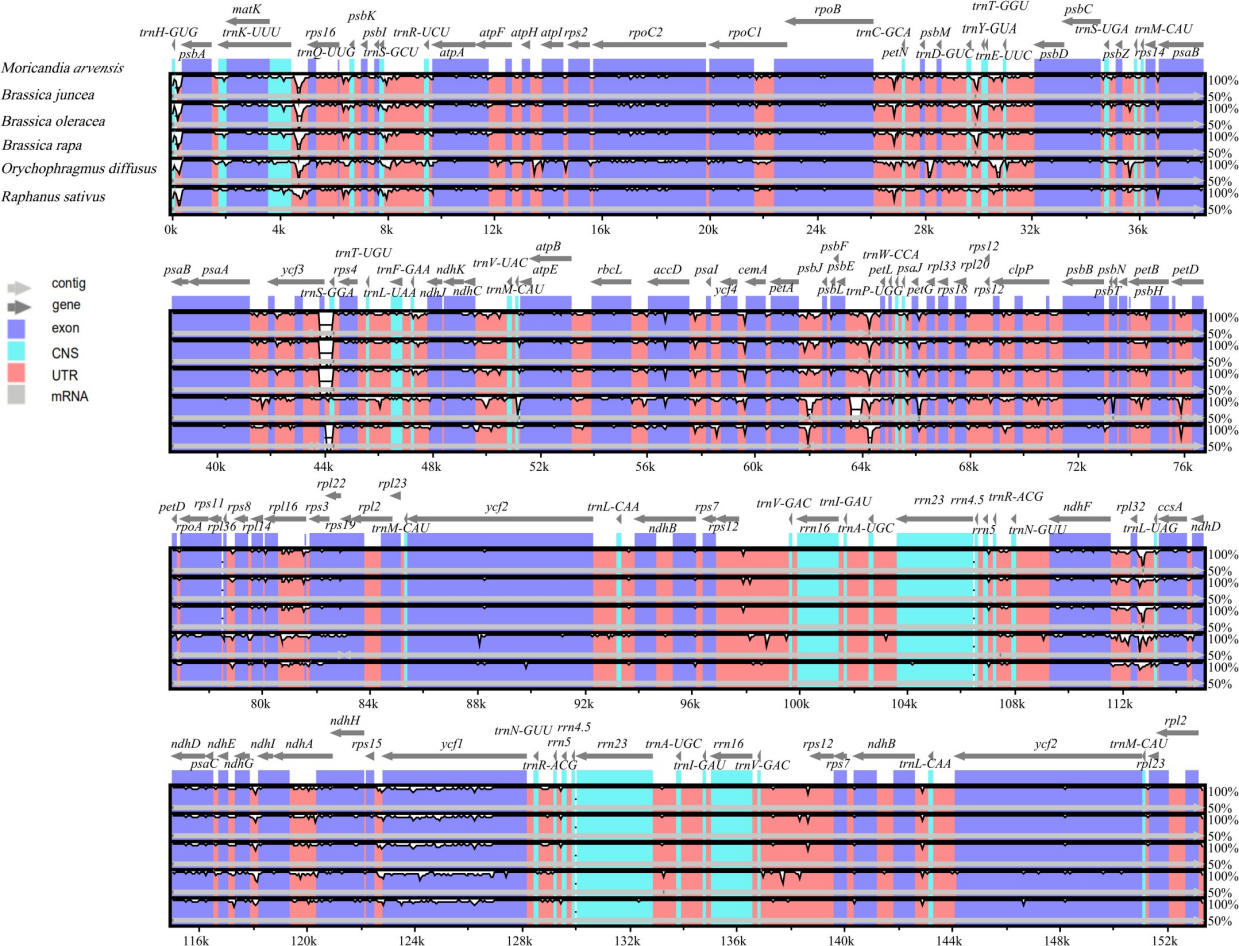
Alignment of the cp genomes of *M*. *arvensis* and five closely related species. The alignment was performed by mVISTA with *M*. *arvensis* as the reference. Local collinear blocks within each alignment are indicated by the same color and linked.

In the comparison of the gene contents of cp genomes among the five related species (*R*. *sativus*, *B*. *juncea*, *B*. *rapa*, *B*. *oleracea*, and *O*. *diffuses*), novel genes were not observed in the *M*. *arvensis* cp genome. The mVISTA program was used to determine whether a change in gene order has occurred in the *M*. *arvensis* cp genome compared to the above mentioned cp genomes using the complete cp genome of *M*. *arvensis* as a reference. The results showed that all selected cp genomes had high similarity (>95%), indicating a high degree of gene synteny, and no gene rearrangement was detected (Fig 2). However, the untranslated regions showed relatively high divergence among the selected cp genomes.

**Fig 3:**
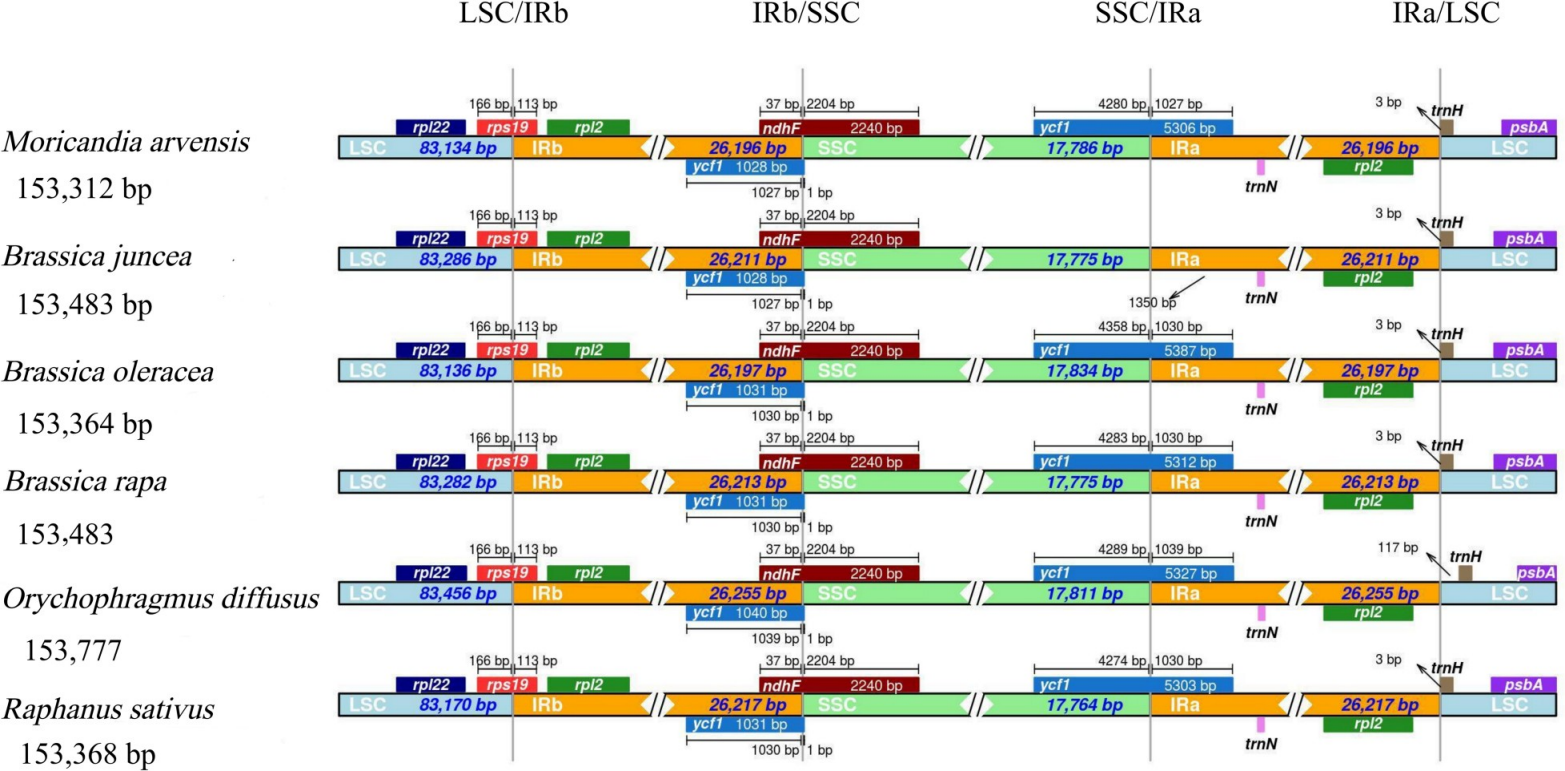
Analysis of the boundaries of LSC/SSC/IR and adjacent genes among six Brassicaceae cp genomes. Sequences of the whole cp genomes *M*. *arvensis* and five closely related cp genomes, including *B*. *rapa*, *B*. *oleracea*, *B*. *juncea*, *R*. *sativus and O*. *diffuses* were downloaded from GenBank.

The LSC/IR/SSC junction regions are believed to be changeable and are considered to be the main factor in structural variations in the cp genomes of higher plants [45]. To detect the divergence of the junction regions between the *M*. *arvensis* cp genome and related species, the LSC/IR/SSC junction regions and adjacent genes in the *M*. *arvensis* cp genome were compared with those in the five above-mentioned species (*R*. *sativus*, *B*. *juncea*, *B*. *rapa*, *B*. *oleracea*, and *O*. *diffuses*). The results showed that the *M*. *arvensis* cp genome was extremely similar to all tested *Brassica* cp genomes, except for the *B*. *juncea* and *R*. *sativus* cp genomes at the junction regions. Differences only occurred in the distance/cover of the *ycf1* gene to the SSC/IRa and IRb/SSC junctions (Fig 3). The *rps19* gene was always located at the LSC/IRB junction in all cp genomes.

### Repeat sequence and SSR loci detection

We found 49 pairs of long repeat sequences ranging from 30 to 69 bp in the *M*. *arvensis* cp genome using REPuter, and we found that these sequences were predominantly composed of palindromic repeats (28 pairs), followed by forward repeats (19 pairs), and reverse repeats (two pairs), but no complementary repeats were observed (Table 3). Most long repeat sequence pairs (67.35%) were found in the same gene or in the same intergenic spacer region (IGS) region. Sixteen long repeat sequence pairs, most of which were belonged to the forward type, were identified in different genes or intergenic spacer (IGS) regions.

**Table 3:** Repeat sequences in the *Moricandia arvensis* cp genome.

No.	Repeat	Type	Repeat 1 Start (Location)	Repeat 2 Start (Location)	Region
1	30	F	106671 *(rrn4*.*5s)*	106703 *(rrn4*.*5s)*	IRA
2	34	F	129713 *(rrn4*.*5s)*	129745 *(rrn4*.*5s)*	IRB
3	34	F	106667 *(rrn4*.*5s)*	106699 *(rrn4*.*5s)*	IRA
4	37	F	97748 *(rps7-trnV-GAC)*	119335 *(ndhA)*	IRA;SSC
5	39	F	43158 *(ycf3)*	97745 *(rps7-trnV-GAC)*	SSC;IRA
6	40	F	148328 *(ycf2)*	148349 *(ycf2)*	IRB
7	42	F	97743 *(rps7-trnV-GAC)*	119330 *(ndhA)*	IRA;SSC
8	47	F	148321 *(ycf2)*	148342 *(ycf2)*	IRB
9	47	F	88057 *(ycf2)*	88078 *(ycf2)*	IRA
10	52	F	38049 *(psaB)*	40273 *(psaA)*	LSC
11	55	F	38046 *(psaB)*	40270 *(psaA)*	LSC
12	58	F	38043 *(psaB)*	40267 *(psaA)*	LSC
13	64	F	38007 *(psaB)*	40231 *(psaA)*	LSC
14	71	F	38013 *(psaB)*	40237 *(psaA)*	LSC
15	73	F	38028 *(psaB)*	40252 *(psaA)*	LSC
16	74	F	38010 *(psaB)*	40234 *(psaA)*	LSC
17	76	F	38025 *(psaB)*	40249 *(psaA)*	LSC
18	79	F	38016 *(psaB)*	40240 *(psaA)*	LSC
19	81	F	38020 *(psaB)*	40244 *(psaA)*	LSC
20	30	P	7725 *(trnS-GCU)*	44261 *(trnS-GGA)*	LSC
21	30	P	61749 *(petA-psbJ)*	61749 *(petA-psbJ)*	LSC
22	30	P	106671 *(rrn4*.*5s)*	129713 *(rrn4*.*5s)*	IRA;IRB
23	30	P	106703 *(rrn4*.*5s)*	129745 *(rrn4*.*5s)*	IRA;IRB
24	34	P	106667 *(rrn4*.*5s)*	129713 *(rrn4*.*5s)*	IRA;IRB
25	34	P	106699 *(rrn4*.*5s)*	129745 *(rrn4*.*5s)*	IRA;IRB
26	37	P	119335 *(ndhA)*	138661 *(trnV-GAC-rps7)*	SSC;IRB
27	38	P	79499 *(rps8-rpl14)*	79499 *(rps8-rpl14)*	LSC
28	39	P	51159 *(trnM-CAU-atpE)*	51159 *(trnM-CAU-atpE)*	LSC
29	39	P	43158 *(ycf3)*	138662 *(trnV-GAC-rps7)*	LSC;IRB
30	40	P	28339 *(petN-psbM)*	28339 *(petN-psbM)*	LSC
31	42	P	296 *(trnH-GUG-psbA)*	296 *(trnH-GUG-psbA)*	LSC
32	42	P	119330 *(ndhA)*	138661 *(trnV-GAC-rps7)*	SSC;IRB
33	44	P	73294 *(psbT-psbN)*	73294 *(psbT-psbN)*	LSC
34	44	P	61981 *(petA-psbJ)*	61981 *(petA-psbJ)*	LSC
35	44	P	76808 *(petD-rpoA)*	76808 *(petD-rpoA)*	LSC
36	45	P	112700 *(rpl32-trnL-UAG)*	112700 *(rpl32-trnL-UAG)*	SSC
37	46	P	9315 *(trnG-UCC-trnR-UCU)*	9315 *(trnG-UCC-trnR-UCU)*	IRA;LSC
38	46	P	209 *(trnH-GUG-psbA)*	209 *(trnH-GUG-psbA)*	LSC
39	46	P	4686 *(matK-rps16)*	4686 *(matK-rps16)*	LSC
40	47	P	64216 *(psbE-petL)*	64216 *(psbE-petL)*	LSC
41	47	P	88057 *(ycf2)*	148321 *(ycf2)*	IRA;IRB
42	47	P	88078 *(ycf2)*	148342 *(ycf2)*	IRA;IRB
43	50	P	55437 *(rbcL-accD)*	55437 *(rbcL-accD)*	LSC
44	50	P	288 *(trnH-GUG-psbA)*	296 *(trnH-GUG-psbA)*	LSC
45	53	P	66075 *(psaJ-rpl33)*	66075 *(psaJ-rpl33)*	LSC
46	53	P	112692 *(rpl32-trnL-UAG)*	112700 *(rpl32-trnL-UAG)*	SSC
47	58	P	64205 *(psbE-petL)*	64216 *(psbE-petL)*	LSC
48	41	R	30823 *(trnE-UUC-trnT-GGU)*	30823 *(trnE-UUC-trnT-GGU)*	LSC
49	47	R	80843 *(rpl16)*	80843 *(rpl16)*	LSC

F: forward repeats; R: reverse repeats; P: palindrome repeats.

Additionally, we identified 51 SSR loci in the *M*. *arvensis* cp genome, comprising 33 mononucleotides, 12 dinucleotides, one trinucleotide, four tetranucleotides, and one hexanucleotide (Table 4). The longest SSR was a 22 bp long mononucleotide repeat located within *ycf1*. Only A-type (12 SSRs) and T-type (21 SSRs) mononucleotide repeats were observed. AT/TA was present in all 12 dinucleotide repeats, and the longest type of dinucleotide repeat was AT (20 bp). The unique trinucleotide repeat was ATT (12 bp), which was found in the intergenic spacer IGS region between *trnT-UGU* and *trnL-UAA*. Three tetranucleotide repeat types (CAAA, TAAA, and ATAG) and one hexanucleotide repeat (GAAAGT) were also detected in the *M*. *arvensis* cp genome. Among the SSR loci, 32 were found in the intergenic spacer IGS regions, accounting for 62.75% of the total SSRs. The remaining SSR loci were distributed in 11 genes, and the pseudogene *ycf1* harbored most SSRs (six SSR loci).

**Table 4:** Distribution of SSRs in the *Moricandia arvensis* cp genome.

Type	Unit	Length	No.	Position on Genoem	Loction
P1	A	10	4	13019–13028	*atpF-atpH*
				29879–29888	*trnD-GUC-trnY-GUA*
				35524–35533	*psbZ-trnG-GCC*
				109311–109320	*ycf1*
		11	2	13753–13763	*atpH-atpI*
				27132–27143	*rpoB-trnC-GCA*
		12	2	122601–122612	*rps15-ycf1*
				137357–137368	*trnV-GAC-rps7*
		13	3	41894–41906	*psaA-ycf3*
				64295–64307	*psbE-petL*
				80068–80080	*rpl14-rpl16*
		14	1	113672–113685	*ccsA*
	T	10	9	25406–25415	*rpoB*
				49134–49143	*ndhK-ndhC*
				50320–50329	*trnV-UAC*
				51211–51220	*trnM-CAU-atpE*
				70416–70425	*clpP*
				78955–78964	*rpl36-rps8*
				80675–80684	*rpl16*
				112043–112052	*ndhF-rpl32*
				127127–127136	*ycf1*
		11	4	17669–17679	*rpoC2*
				29564–29574	*psbM-trnD-GUC*
				45504–45514	*rps4-trnT-UGU*
				123187–123197	*ycf1*
		12	4	74235–74246	*petB*
				99079–99090	*rps7-trnV-GAC*
				124947–124958	*ycf1*
				125265–125276	*ycf1*
		13	3	65602–65614	*trnP-UGG-psaJ*
				77163–77175	*rpoA*
				81549–81561	*rpl16*
		22	1	124755–124776	*ycf1*
P2	AT	10	6	26644–26653	*rpoB-trnC-GCA*
				35661–35670	*psbZ-trnG-GCC*
				107443–107452	*trnR-ACG-trnN-GUU*
				120298–120307	*ndhA*
				128995–129004	*trnN-GUU-trnR-ACG*
				142979–142988	*ndhB-trnL-CAA*
		14	1	13471–13484	*atpH-atpI*
		20	1	30833–30852	*trnE-UUC-trnT-GGU*
	TA	10	4	19041–19050	*rpoC2*
				62092–62101	*petA-psbJ*
				93458–93467	*trnL-CAA-ndhB*
				111594–111603	*ndhF-rpl32*
P3	ATT	12	1	45957–45968	*trnT-UGU-trnL-UAA*
P4	CAAA	12	1	28186–28197	*petN-psbM*
	TAAA	12	2	45780–45791	*trnT-UGU-trnL-UAA*
	ATAG	12	1	111356–111367	*ndhF*
P6	GAAAGT	18	1	56632–56649	*accD*

### Analysis of codon usage bias

The sequences of 79 protein-coding genes generated 25,447 codons. Among them, the leucine (Leu) codon was the most common, accounting for 10.46% of total codons, followed by those encoding isoleucine (8.67), whereas the encoding cysteine (Cys) showed the lowest frequency of only 1.21% (Table 5). Additionally, the relative synonymous codon usage (RSCU) value was calculated to assess the codon usage bias of the *M*. *arvensis* cp genome. The RSCU values of 30 codons were > 1, indicating that they are preferentially used in the *M*. *arvensis* cp genome. The UUA codon, encoding leucine, showed the highest usage bias, with an RSCU value of 2.04. Among the preferentially used codons, all codons ended with U (16 of 30) or A (13 of 30), except for UUG, which encodes leucine.

**Table 5:** Summary of codon usage and amino acids patterns of *Moricandia arvensis* cp genome.

Codon	Number	Amino acids	Ratio of Codon	RSCU	No.	Ratio
GCA	366	Ala	1.44%	1.11	1319	5.18%
GCC	200		0.79%	0.61		
GCG	144		0.57%	0.44		
GCU	609		2.39%	1.85		
AGA	463	Arg	1.82%	1.82	1528	6.00%
AGG	153		0.60%	0.60		
CGA	349		1.37%	1.37		
CGC	104		0.41%	0.41		
CGG	123		0.48%	0.48		
CGU	336		1.32%	1.32		
AAC	284	Asn	1.12%	0.46	1245	4.89%
AAU	961		3.78%	1.54		
GAC	195	Asp	0.77%	0.38	1015	3.99%
GAU	820		3.22%	1.62		
UGC	75	Cys	0.29%	0.49	309	1.21%
UGU	234		0.92%	1.51		
CAA	707	Gln	2.78%	1.55	911	3.58%
CAG	204		0.80%	0.45		
GAA	1029	Glu	4.04%	1.51	1359	5.34%
GAG	330		1.30%	0.49		
GGA	706	Gly	2.77%	1.66	1705	6.70%
GGC	167		0.66%	0.39		
GGG	276		1.08%	0.65		
GGU	556		2.18%	1.30		
CAC	147	His	0.58%	0.50	593	2.33%
CAU	446		1.75%	1.50		
AUA	710	Ile	2.79%	0.97	2206	8.67%
AUC	398		1.56%	0.54		
AUU	1098		4.31%	1.49		
CUA	365	Leu	1.43%	0.82	2662	10.46%
CUC	162		0.64%	0.37		
CUG	162		0.64%	0.37		
CUU	558		2.19%	1.26		
UUA	905		3.56%	2.04		
UUG	510		2.00%	1.15		
AAA	1107	Lys	4.35%	1.53	1451	5.70%
AAG	344		1.35%	0.47		
AUG	555	Met	2.18%	1.00	555	2.18%
UUC	490	Phe	1.93%	0.64	1521	5.98%
UUU	1031		4.05%	1.36		
CCA	285	Pro	1.12%	1.12	1022	4.02%
CCC	190		0.75%	0.74		
CCG	136		0.53%	0.53		
CCU	411		1.62%	1.61		
AGC	121	Ser	0.48%	0.37	1956	7.69%
AGU	394		1.55%	1.21		
UCA	393		1.54%	1.21		
UCC	286		1.12%	0.88		
UCG	192		0.75%	0.59		
UCU	570		2.24%	1.75		
UAA	41	TER	0.16%	1.86	66	0.26%
UAG	17		0.07%	0.77		
UGA	8		0.03%	0.36		
ACA	406	Thr	1.60%	1.25	1300	5.11%
ACC	228		0.90%	0.70		
ACG	143		0.56%	0.44		
ACU	523		2.06%	1.61		
UGG	430	Trp	1.69%	1.00	430	1.69%
UAC	176	Tyr	0.69%	0.37	951	3.74%
UAU	775		3.05%	1.63		
GUA	489	Val	1.92%	1.46	1343	5.28%
GUC	165		0.73%	0.49		
GUG	190		0.75%	0.57		
GUU	499	Ala	1.96%	1.49		

### Analysis of Ka and Ks substitution rate

The Ka/Ks ratio has been extensively used to assess how genomic evolution and selection pressure affect genes [41, 46]. The ratio of Ka/Ks < 1, Ka/Ks = 1, and Ka/Ks > 1 indicate genes that underwent purifying, neutral, and positive selections, respectively [40]. To determine whether the Ka/Ks ratio provides clues for the evolution of photosynthesis, we calculated the Ka/Ks ratios of 77 homologous coding genes (S3 Table) between the cp genome of *M*. *arvensis* and cp genome of the five related species (*R*. *sativus*, *B*. *juncea*, *B*. *rapa*, *B*. *oleracea*, *and O*. *diffuses*). The results showed that almost all selected genes had Ka/Ks values < 1 (except *petD* in the comparison of *B*. *juncea* vs. *M*. *arvensis* and *ycf2* in the comparison of *R*. *sativus* vs. *M*. *arvensis*). Some genes associated with “Subunits of ATP synthase” (*atpB*), “Subunit of acetyl-CoA” (*accD*), “Subunits of NADH-dehydrogenase” (*ndhG* and *ndhE*), and “Self-replication” (*rps12* and *rpl16*), generally showed relatively higher Ka/Ks values (> 0.5) than those of other genes, indicating that these genes have likely undergone relatively higher purifying selection pressure.

### Phylogenetic analysis

**Fig 4:**
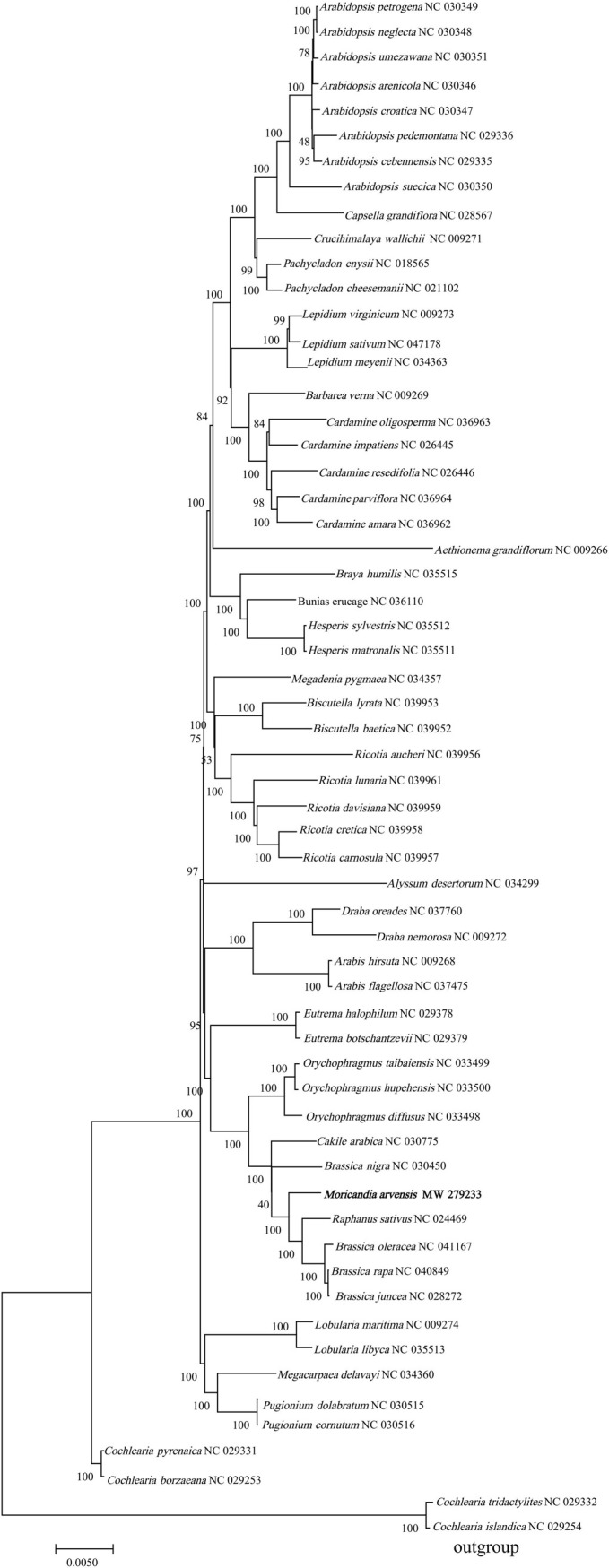
Phylogenetic analysis of 61 Brassicaceae species based on the shared common protein-coding sequence. The evolutionary history was inferred using the Maximum Likelihood method based on the Tamura-Nei model. The bootstrap values are shown next to the nodes. The initial tree(s) for the heuristic search were obtained automatically by applying the Neighbor-Join and BioNJ algorithms to a matrix of pairwise distances estimated by the Maximum Likelihood (ML) approach, and then selecting the topology with the highest log value. The tree is drawn to scale, with branch length measured by the number of substitutions per site.

To determine the genealogical position of *M*. *arvensis* within Brassicaceae and to determine whether *M*. *arvensis* with C3-C4 characters underwent a unique evolutionary origin event, we downloaded cp genomes of 60 Brassicaceae species from the NCBI to construct a phylogenetic tree. The phylogenetic tree (Fig 4) demonstrated that *M*. *arvensis* was not clustered into an independent branch, but constituted a subclade with cultivated *Brassica* species (*B*. *oleracea*, *B*. *rapa*, and *B*. *juncea*) and *R*. *sativus*, indicating that it is unlikely that *M*. *arvensis* was involved in independent evolutionary origin events. Sixty branches were generated in the phylogenetic tree. Among them, 55 branches were supported by node values > 50%, and 48 branches had node values > 95%. *Cochlearia* species formed an outgroup; however, the four tested *Cochlearia* species did not constitute a single subclade, suggesting that *Cochlearia* species radiated in different directions.

## Discussion

C4 plants have been studied extensively because of their effective photosynthetic carbon fixation efficiency, accounting for up to 25% of the Earth’s primary productivity [14, 47]. These C3-C4 intermediate plants are believed to be a bridge during C3–-C4 evolution [48, 49]. Understanding the cp genome of such C3-C4 intermediates will likely provide essential information on the evolution of photosynthesis. The present study, which reports the complete plastome sequence of M. arvensis confirmed, that joint application of two sequencing platforms, PacBio with long reads and Illumina with short reads, provide efficient and reliable approach for assembling and annotation of chloroplast genomes [17, 50].

The *M*. *arvensis* cp genome had a typical quadripartite structure of 153,312 bp, which is comparable with the size of other Brassicaceae cp genomes [17, 51–53]. The gene content, gene order, and LSC/IR/SSC junction regions of the *M*. *arvensis* cp genome were highly conserved compared to those of related C3 species, indicating that no significant change has occurred in the cp genome during C3 to C3-C4 intermediate evolution. A recent study aimed at deciphering the cp genomes of C3, Kranz type C4 and single cell C4 photosynthetic members in Chenopodiaceae also showed that the cp genomes of C3, C4, and single cell C4 species had similar organizations, gene orders, and contents [54].

RLSB binding of chloroplastic *rbcL* mRNA is associated with C3 to C4 evolution in *Flaveria* [14]. However, *rbcL* did not reveal high Ka or Ks ratios in the *M*. *arvensis* cp genome (S3 Table) compared to those in related C3 species. In contrast, the genes associated with “Subunits of ATP synthase” (*atpB*), “Subunits of NADH-dehydrogenase” (*ndhG* and *ndhE*), and “Self-replication” (*rps12* and *rpl16*) had relatively higher Ka/Ks values than those of other genes, indicating that these genes have likely accompanied the evolution of C3 to C3-C4 intermediates.

Phylogenetic studies have demonstrated that C3-C4 intermediates are more closely related to their C4 relatives than to their C3 relatives in families comprising C3, C3-C4 intermediate, and C4 (C4-like) species [24]. However, C4 plants are not distributed equally in the plant kingdom [55], and they have not been identified in Brassicaeae. To determine whether *M*. *arvensis* underwent a special evolutionary origin event compared to its C3 relatives, a phylogenetic tree was constructed based on common coding sequences. *M*. *arvensis* was not clustered into an independent branch, but it constituted a subclade with the cultivated *Brassica* species and *R*. *sativus*. This result is in accordance with the results of the phylogenetic analysis carried out by Warwick and Sauder [56] based on cp *trnL* intron sequences. A recent phylogenetic study of different *Moricandia* lines based on ITS data revealed a close relationship between C3-C4 intermediates and their C3 siblings [8]. These results indicate that the C3-C4 intermediate *M*. *arvensis* did not evolve through an independent evolutionary origin event.

## Conclusion

In the present study, we obtained a well-annotated cp genome of *M*. *arvensis* with a C3-C4 intermediate character. We did not detect conspicuous genomic divergence in the *M*. *arvensis* cp genome compared to that of other related C3 species. However, the Ka/Ks analysis showed that some genes associated with photosynthesis had high Ka/Ks values compared with the genes of several related C3 species, indicating that these genes have likely accompanied the evolution of C3 to C3-C4 intermediates. Our phylogenetic analysis demonstrated that *M*. *arvensis* was clustered into a subclade with cultivated *Brassica* species and *R*. *sativus*, indicating that *M*. *arvensis* was not involved in an independent evolutionary origin event. These results will provide guidance for utilizing *M*. *arvensis* as a resource for improving photosynthesis in cultivated *Brassica* species and enable the collection of more data regarding the evolutionary path from C3 to C4. In future studies, deciphering nuclear genomic information will be vital to reveal the key steps in the evolution from C3 to C3-C4 intermediates.

## Supporting information

S1 Table Synonymous (Ks) and non synonymous (Ka) substitution rate of pairwise comparisons of 77 protein coding genes between *Moricandia arvensis* and other five closed related species. (XLSX)

S2 Table List of the cp genome of 60 Brassicaceae species used for phylogenetic analysis. (XLSX)

S3 Table List of the 66 shared genes used to construct the phylogenetic tree. (XLSX)

S4 Table Summary of *de novo* sequencing of cp genome of *Moricandia arvensis*. (XLSX)

## Abbreviations

Cp: chloroplast
IR: inverted repeat
LSC: large single copy
SSC: small single copy
rRNA: ribosomal RNA
tRNA: transfer RNA
SSR: simple sequence repeat
RLSB: the nuclear-encoded rbcL RNA S1 binding domain protein
RSCU: the relative synonymous codon usage
ML: maximum likelihood
Ka: non-synonymous
Ks: synonymous
